# Optimizing skin disease diagnosis: harnessing online community data with contrastive learning and clustering techniques

**DOI:** 10.1038/s41746-024-01014-x

**Published:** 2024-02-08

**Authors:** Yue Shen, Huanyu Li, Can Sun, Hongtao Ji, Daojun Zhang, Kun Hu, Yiqi Tang, Yu Chen, Zikun Wei, Junwei Lv

**Affiliations:** 1 Simulation of Complex Systems Lab, Department of Human and Engineered Environmental Studies, Graduate School of Frontier Sciences, The University of Tokyo, Chiba, Japan; 2 Shanghai Beforteen AI Lab, Shanghai, China; 3 Institution of Aix-marseille, Wuhan University of Technology WHUT, Wuhan City, China; 4 Shanghai Business School No. 6333, Oriental Meigu Avenue, Shanghai, China; 5 The third affiliated hospital of CQMU, Chongqing, China

**Keywords:** Physical examination, Technology

## Abstract

Skin diseases pose significant challenges in China. Internet health forums offer a platform for millions of users to discuss skin diseases and share images for early intervention, leaving large amount of valuable dermatology images. However, data quality and annotation challenges limit the potential of these resources for developing diagnostic models. In this study, we proposed a deep-learning model that utilized unannotated dermatology images from diverse online sources. We adopted a contrastive learning approach to learn general representations from unlabeled images and fine-tuned the model on coarsely annotated images from Internet forums. Our model classified 22 common skin diseases. To improve annotation quality, we used a clustering method with a small set of standardized validation images. We tested the model on images collected by 33 experienced dermatologists from 15 tertiary hospitals and achieved a 45.05% top-1 accuracy, outperforming the published baseline model by 3%. Accuracy increased with additional validation images, reaching 49.64% with 50 images per category. Our model also demonstrated transferability to new tasks, such as detecting monkeypox, with a 61.76% top-1 accuracy using only 50 additional images in the training process. We also tested our model on benchmark datasets to show the generalization ability. Our findings highlight the potential of unannotated images from online forums for future dermatology applications and demonstrate the effectiveness of our model for early diagnosis and potential outbreak mitigation.

## Introduction

Skin diseases pose a significant challenge in China. There are up to 240 million dermatological visits per year, among which 80% are for skin diseases beyond melanoma. Nevertheless, the uneven distribution of healthcare resources and a shortage of dermatologists can lead to misdiagnosis and rising medical costs^1^. To improve the prognosis and reduce social costs, accurate and convenient diagnosis of skin diseases is critical. Artificial intelligence (AI) has shown great potential in dermatology due to the widespread use of photography in diagnosis^2,3^. However, most AI applications focus on benign and malignant lesion diagnosis^4^, leaving the potential of AI for a broader range of skin diseases largely unexplored.

Diagnosing skin diseases with machine learning methods^5–7^ and its deep learning branch using convolutional neural networks (CNNs)^8–11^ based on photographs has received much attention. While high-quality images are critical for training AI models, the labor costs associated with collecting these images can be prohibitively expensive. Fortunately, recent advancements in self-supervised contrastive learning offer a solution. These methods enable the pre-training of models using vast amounts of unlabeled or non-strictly labeled images and have shown outstanding performance in various tasks^12–15^. There are prior works exploring contrastive learning in dermatological diagnosis, emphasizing its capability to extract consistent representations and enhance generalizability and diagnostic accuracy^16,17^. For instance, FairDisCo^18^ applied contrastive learning with additional network branches to enhance fairness across different ethnics. Ref. ^19^ introduced federated contrastive learning for dermatological disease diagnosis via on-device learning. Other studies have indicated that utilizing contrastive learning methods to combine multi-level features of skin lesion images can enhance the accuracy of diagnosing skin diseases^20,21^. However, most of these models are trained and tested on professional benchmark datasets. This can pose challenges when applying models trained on professional images to non-professional ones^22,23^. Benchmark datasets are typically captured in controlled medical research settings and focus on diseases with high medical significance, such as malignancies. Consequently, there’s a significant gap between these dataset’s distributions and the prevalence of common skin diseases in daily life. Also, the diversity in image capture settings restricts the generalizability of these models in society. To bridge this gap from the source data, the abundant unlabeled and coarse-labeled skin image data from online forums has come to our view. Traditionally, their unscreened and unannotated nature renders them unsuitable for traditional AI training, which calls for further exploration.

In this study, we present a deep-learning framework that leverages vast amounts of unannotated and coarse-labeled dermatology images from online sources. We employ a three-stage classification algorithm based on contrastive learning. In the pre-training stage, the model learns feature representations from unlabeled images. Our pre-trained model can be fine-tuned to downstream tasks with better performance compared with baseline models trained on general dataset. To reduce the effect of incorrect labels in the fine-tuning using Internet-sourced images, we propose a filtering approach using features extracted with unsupervised model and clustering approaches. This approach not only reduces training costs but also improves the model’s generalization ability for online diagnosis scenarios. Additionally, our method allows for easy fine-tuning on novel categories with limited standardized images, reducing data collection time and labor costs. We demonstrate this with an early warning system for monkeypox. Also, we have tested the performance of our model on benchmark datasets to show the generalize ability of our model when facing images from different ethnicities. In summary, our work reveals a new direction for dermatology AI research, leveraging unannotated and coarse-labeled internet-derived image data and contrastive learning to develop deep learning models for skin disease diagnosis. This approach has the potential to revolutionize dermatology, offering a more efficient and cost-effective method for diagnosing a wide range of skin diseases and ultimately improving patient outcomes.

## Results

### Evaluation of pre-trained models

To evaluate the performance of our pre-trained model, we fine-tuned it using the entire coarse-labeled training set of 0.13 million images. To assess the efficacy of our model, we compared our results (denoted as ‘Derm’) with those obtained using a published pre-trained model trained with ImageNet (denoted as ‘ImageNet’). At the same time, we note that some work has pointed out that knowledge from ImageNet can speed up convergence, improve the generalization ability and performance when facing new problem domains^17,24^. Therefore, we designed two additional pre-training experiments to explore the role of ImageNet in understanding online dermatology images. The first experiment involved mixing the images from ImageNet and our dermatology images for pre-training (denoted as ‘ImageNet+Derm’). The second experiment pre-trained the model on dermatology images initialized with self-supervised ImageNet weights (denoted as ‘ImageNet→Derm’). All four pre-trained models were fine-tuned under the same setting with 0.13 million coarse-labeled images. The results are presented in Table 1. Our top-1 diagnostic accuracy on the test set increased from 42.05% to 45.05% when pretrained sorely on dermatology images, indicating a notable improvement in performance. These findings suggest that unlabeled skin disease data available on the Internet, even without standardized sampling and labeling processes, holds great potential in the field of skin disease diagnosis. It is noteworthy that simply combining ImageNet and dermatology image data for pre-training shows only a marginal increase in top-1 accuracy for dermatology classification. Initializing with ImageNet model weights brings greater gains than simply mixing the two datasets. However, it should also be noted that these pre-training approaches incur a greater training cost than solely working on the dermatology dataset or the ImageNet dataset. The increase in training cost primarily arises from the dataset expansion. We conducted pre-training for each method using 64 RTX 3090 GPUs. In the ‘ImageNet+Derm’ configuration, the training duration (approximately 12 h per 100 epochs) almost doubles compared to the ‘Derm’ setup (approximately 5 h and 45 min per 100 epochs). While in this study, we utilized publicly available models trained on ImageNet, resulting in a comparable computational cost for ‘ImageNet→Derm’ as with the ‘Derm’ setup, it’s crucial not to overlook the additional time required to acquire model weights trained on ImageNet if different network architectures were to be employed. We attribute the modest gains from the ‘ImageNet→Derm’ approach to catastrophic forgetting, where the model loses previously acquired knowledge from ImageNet when exposed to new, unlabeled data. Additionally, the high prevalence of label noise within internet-sourced dermatology images likely hinders the model’s ability to learn accurate representations in fine-tuning without selective filtering.

**Table 1:** Performance comparison among different pre-training strategies.

Pre-training Dataset	Top-1 Accuracy (%)	Top-3 Accuracy (%)	Top-5 Accuracy (%)	AUC
ImageNet	42.05	64.42	74.36	0.859
Derm	45.05	65.13	74.77	0.872
ImageNet+Derm	45.12	66.29	75.86	0.866
ImageNet→Derm	46.13	67.03	76.69	0.874

Four pre-training strategies were adopted: pretrained purely on ImageNet dataset, pretrained purely on online dermatology dataset, pretrained on a mixture of ImageNet and online dermatology dataset, pretrained on online dermatology dataset initialized with ImageNet weights. We assessed the performance of these pre-training models on our test set through fine-tuning using the 0.13 million coarse-labeled images. Despite a marginal increase, the performance gain from combining the ImageNet dataset in pre-training is not as significant as our filtering approach, and it necessitates additional computational resources.

### Effect of filtering coarse-labeled data

We acknowledge the challenge posed by noisy labeling in these coarse-labeled images during the fine-tuning stage. To address this, we filtered the training set using a validation set of 20 images per disease based on feature distance obtained by pre-trained model. This approach reduced the number of training images from 0.13 million to approximately 30000, but our model’s top-1 diagnostic accuracy improved from 45.05% to 46.61%, and top-3 accuracy increased from 65.13% to 68.48%, which also surpass the gains brought by pre-training on models initialized with ImageNet model weights, indicating the necessity of our filtering methodology. These findings indicate that using a larger validation set to obtain a more comprehensive description of the clusters per disease may lead to more effective filtering results.

**Fig. 1:**
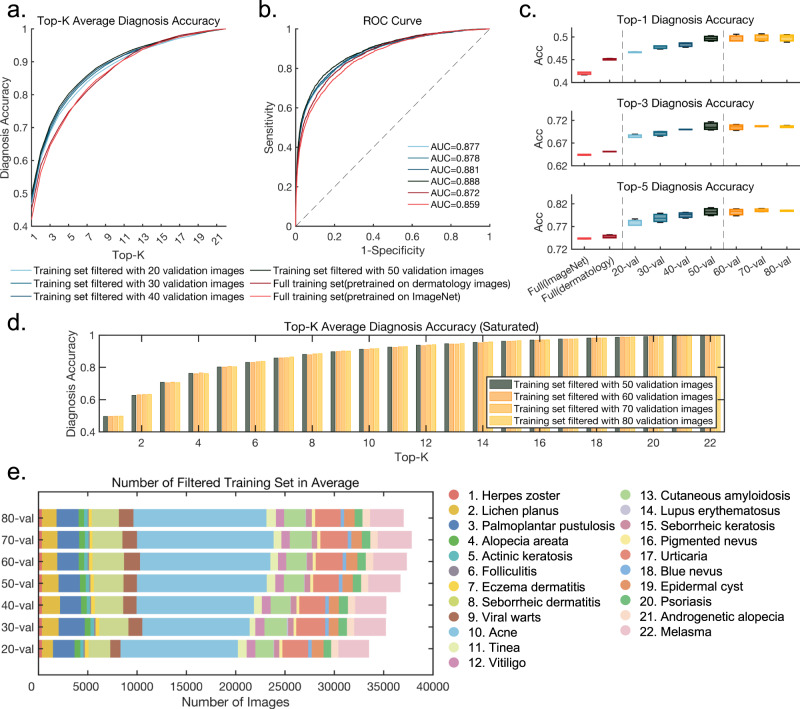
Model performance compared with the published baseline using the filtered training set. **a**, **b** show the top-k diagnosis accuracy and ROC curve of our model. We pre-trained our model using unannotated images collected from the Internet and then fine-tuned it on the full coarse labeled training set. Our top-1 diagnostic accuracy on the test set increased from 42.05% to 45.05% and the AUC of the ROC curve increased from 0.859 to 0.872. After filtering potential noisy labels using validation images, the performance improved as the number of validation images increased. When there were 50 validation images per category, the top-1 accuracy reached 49.64%. **c** Boxplot showing the performance of three trials using different subset of validation images. Boxes represent the median costs and interquartile range. Whiskers extend to the farthest data points. ANOVA analysis showed that our model’s performance was significantly better than the baseline, and that different validation sets used for filtering did not produce statistically significant differences (*p* = 0.77). **d** Top-k diagnosis accuracy improvement of our model saturates when the number of validation images reaches 50 per category, suggesting that 50 validation images per category are sufficient for the filtering process. **e** Number of images after filtering averaged over three trials did not vary too much when changing the number of validation images, especially when the number of validation images reached 50 per category, indicating the estimated center of each cluster tends to be stable with more validation images.

To develop a flexible model adaptable to various diseases, reducing the amount of labeled data can significantly decrease training time and costs. However, using too little data may not effectively capture the feature clusters of a disease. To further explore this issue, we randomly selected subsets of 20, 30, 40, 50, 60, 70, and 80 images from the 80 validation images collected for each disease to examine the impact of the size of the validation set on filtering the coarse-labeled training data and the model’s performance. To ensure test reproducibility, we conducted three trials using different random seeds to select subsets of the validation dataset and fine-tune the model. The final top-k diagnostic accuracy is presented in Fig. 1a. The average top-1 accuracy after filtering images based on 20, 30, 40, and 50 validation samples over the three trials was 46.61%, 47.77%, 48.32%, and 49.64% respectively, indicating significant improvement compared to the baseline of 42.05%. The ROC curve, as Fig. 1b shows, also indicates an improvement in the performance when the validation samples increased. By ANOVA, we are unable to statistically consider the data from the three trials to be significantly different (*p* = 0.77), while statistically indicating that the model performance is significantly higher than the baseline of 42.05% (all *p*-values much less than 0.01) as shown in Fig. 1c. Furthermore, performance improved gradually as the number of validation samples increased, likely due to a more precise description of each cluster center with a larger validation set. However, the improvement in subsequent models was relatively low when the number of validation samples exceeded 50. As shown in the Fig. 1d, the average top-1 accuracy of filtering images based on 60,70,80 validation samples were 49.61%, 49.77%, and 49.79% respectively, while the rest of the top-k accuracy also remained almost the same. We also used our proposed filtering approach with 50 validation images per disease on top of the ‘ImageNet->Derm’ pretrained model and achieved an average top-1 accuracy of 50.44%. Compared with fine-tuning with the whole coarse-labeled training set (46.13%), our filtering approach gave a 4.31% increase, which further proved the effectiveness of our filtering strategy across different pre-training baselines. We counted the average number of images per category after filtering with different number of validation images as Fig. 1e shows. Generally, the number of remaining training sets did not vary too much, especially when the number of validation images reached 50 per category. Intuitively, we think that the estimated cluster centers differ more from the actual cluster centers when there are fewer validation images, thus causing greater bias when filtering the training set by Euclidean distance. Therefore, the estimated center of each cluster tends to be stable with more validation images.

**Fig. 2:**
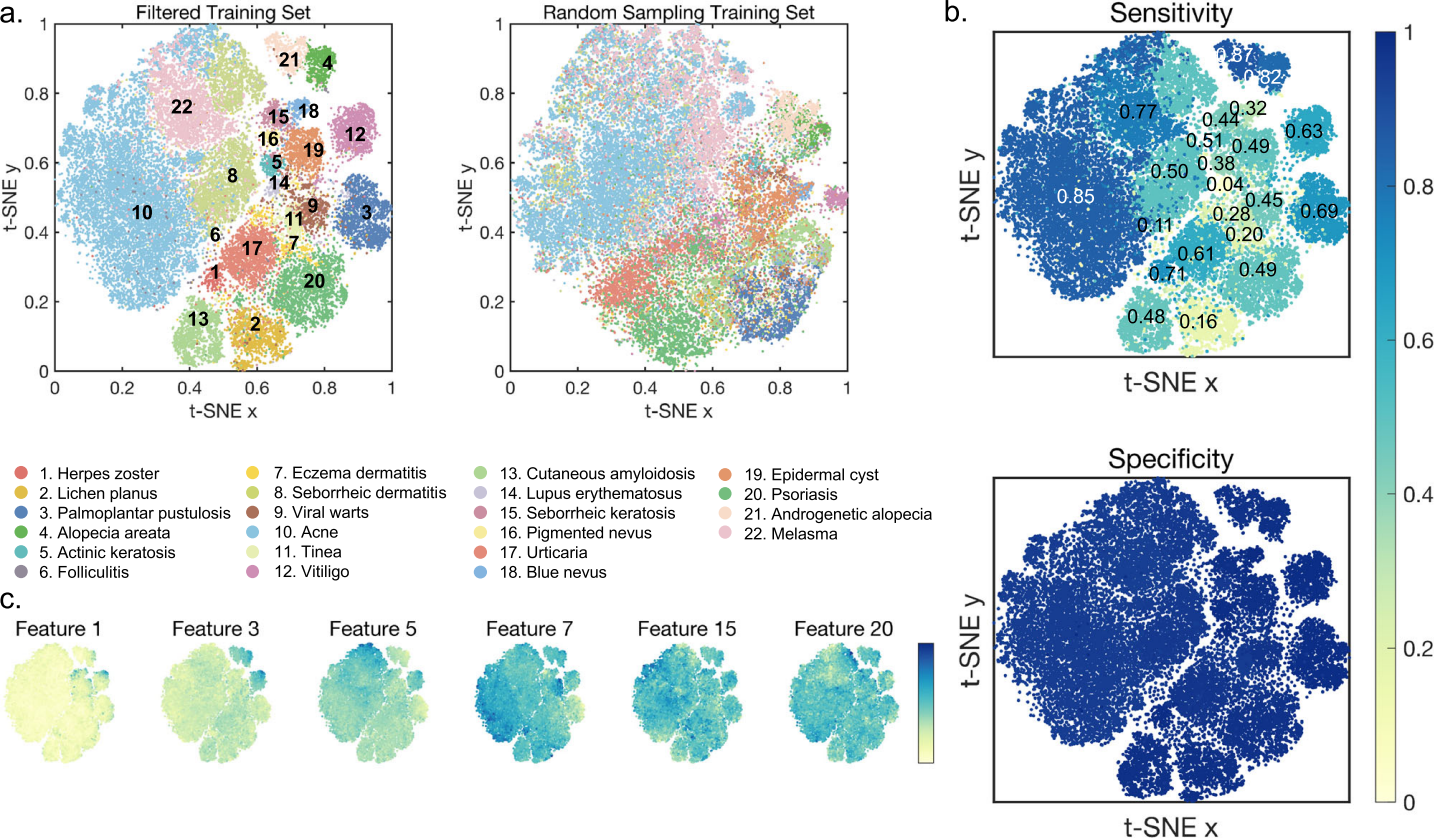
Feature representation of the 22 diseases included in our study. **a** t-SNE plots of the filtered training set with 50 validation images per category colored by disease categories, demonstrating the effectiveness of our approach in capturing the distinct features of each disease. For comparison, a subset of images of the same number is randomly selected from the coarse labeled training set. **b** Average sensitivity and specificity for each disease of 3 trials. Specificity of each category are all over 0.94 but sensitivity varied considerably. Generally, sensitivity would be higher if the cluster was relatively far from the center of other diseases with fewer surrounding clusters. **c** Expression levels of some host features. The levels of these characteristics are associated with the types of skin diseases and affected areas of the skin.

To illustrate the effectiveness of our filtering method, we used t-SNE to generate a scatter plot of the cluster distribution of the remaining training set after filtering with 50 validation images per category. We also randomly sampled the same number of images from the original training set to draw a scatter plot for comparison, as shown in Fig. 2a. While t-SNE may cause some deformation in the appearance and distances of clusters, it still allows for a rough idea of the relative position and coverage of each disease in the feature space. Our results indicate that the selected training set after clustering and filtering using features obtained by the pre-trained model displays clearer boundaries for each cluster, and the relative location of each cluster corresponds to dermatologists’ knowledge. For example, in the upper right corner of the scatter plot, two isolated clusters representing androgenic alopecia and alopecia areata can be observed, which are similar yet distinct from other diseases. The discernible clustering of diverse skin conditions indicates that the features employed in our analysis can capture distinct attributes that are relevant to each disorder. In Fig. 2b we mapped the average top-1 specificity and sensitivity of each disease to the scatter plot. All the specificity were over 0.94, but the sensitivities displayed considerable variation. Generally, diseases with clusters relatively far from the center of other diseases and with fewer surrounding clusters exhibited higher sensitivity. For instance, the sensitivity of androgenetic alopecia, alopecia areata, acne, and melasma were 0.87, 0.82, 0.85, and 0.77, respectively. These diseases are empirically more typical and are easier for the physician to diagnose based on the image alone. Conversely, diseases with clusters closer to the overall center of other diseases and with more surrounding clusters demonstrated poorer sensitivity. For example, lupus erythematosus and eczema dermatitis only got 0.04 and 0.20. These diseases often lack typical lesion characteristics, and the physicians also require additional information to make accurate diagnoses. We present the expression levels of certain host features in Fig. 2c to provide a better understanding of the selected training set. The spatial distribution of selected features highlights the likelihood that these characteristics are linked to specific types of skin diseases and affected areas of the skin. When we randomly selected several images from both the filtered training set and the excluded images and mapped them onto the scatter plots, it can be observed that the retained images generally exhibit typical skin lesion characteristics of their respective diseases, while the excluded images tend to be farther from the cluster centers and are mostly identifiable as label errors. It is important to note, however, that there are three scenarios in which images may have been excluded from the training set. Firstly, images containing more than one skin disease, with the coarse label failing to become the primary focus. Secondly, atypical skin lesions, such as alopecia areata on the eyebrows, also have a high chance of being far away from the typical cluster. And thirdly, skin diseases under treatment, where recovery or medication will also change the appearance of lesions. These exclusions could potentially lead to a decreased recognition capability of our model for atypical skin lesions, even though they only account for a small proportion of the collected skin disease images. While it is generally believed that more labeled data leads to better model performance, our experiments demonstrate that images with correct knowledge and distinct features are more likely to help the model learn diagnostic criteria than a large amount of data with ambiguous or incorrect labels.

### Transfer learning to monkeypox detection

**Fig. 3:**
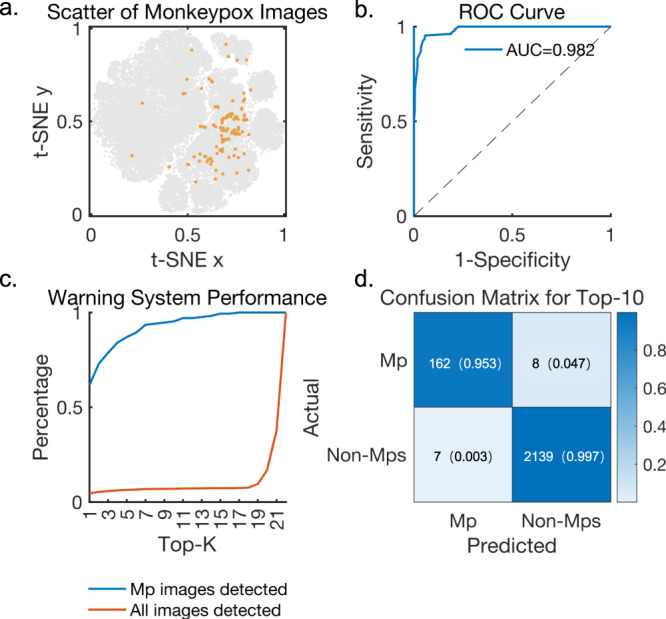
Results of downstream tasks with monkeypox images added. **a** Scatter of monkeypox images. most monkeypox image projections are located near viral warts and tinea. **b** ROC curve of the fine-tuned model. We fine-tuned the model with the filtered training set and 50 extra monkeypox images to act as a warning system for monkeypox, testing on 170 monkeypox images and 2146 skin disease-related images. **c** Performance of the monkeypox warning system at each confidence level. Mp refers to monkeypox images. Our model detected 61.76% of monkeypox images at top-1 level. At the top-10 confidence level, 95.29% of monkeypox images were successfully diagnosed, with highly suspected images accounting for only 6.99% of the total images. **d** Confusion matrix at the top-10 confidence level (Sensitivity = 0.953, Specificity = 0.997).

Figure 1d may suggest that for a new disease, 50 cases may be needed to describe the clusters based on the pre-trained contrastive model provided. Besides, at the top-10 confidence level, the diagnosis accuracy already reached 90%. Therefore, adapting our model to an early warning system for a new disease may no longer require a large amount of image data specific to that disease, resulting in a significant improvement in both training speed and overall cost. We expect the model to identify potential risk images from a large number of uploaded data by internet users and provide early warning signals for emerging diseases. We demonstrate this in the experiment of monkeypox, a rare disease primarily affecting dark-skinned individuals. We fine-tuned our model on 50 monkeypox images added to the selected coarse labeled training set and tested it on a mixed set of 2146 common skin disease images and 170 monkeypox images collected from the internet. For images with low diagnostic confidence, we classified them as ‘others’. In order to ensure suspected patients receive proper diagnostic evaluation, it is important to have a low false negative rate in a primary hospital or internet scenario. Therefore, including a high ratio of monkeypox images in the warning system while blocking unrelated diseases is crucial. Relevant results are shown in Fig. 3. We projected the image features of monkeypox onto the feature representation map of the previously analyzed 22 skin diseases as Fig. 3a shows. Due to the limited amount of image data included, the resulting cluster appears relatively loose. Nevertheless, it is evident that, apart from a few cases that may be attributed to mislabeled images or distinct location-specific features (such as skin lesions near hair), most monkeypox image projections are located near viral warts and tinea. This observation aligns with our understanding of the characteristics associated with monkeypox. The ROC curve for monkeypox is shown in Fig. 3b, with an AUC of 0.982. We counted the images that were diagnosed as monkeypox along with the confidence level as Fig. 3c shows. Our experiments show that when the confidence level is ranked in the top 10, 95.29% of monkeypox images are diagnosed, with suspected images accounting for only 6.99% of the total images, the confusion matrix of which is shown in Fig. 3d. The sensitivity was 0.953 and specificity was 0.997. Furthermore, our model detected 61.76% of monkeypox images at top-1 level, which surpasses 15 of the 22 regular skin diseases. This suggests that the model has promising performance in identifying rare and emerging diseases while utilizing a small volume of training data. In addition, we observed that images classified as ‘others’ in our experiments, mostly consisted of skin diseases that were not included in the training process like fungal skin infections. This may be due to the highly distinctive nature of these skin lesions, which neither resemble typical monkeypox lesions nor bear similarities to any of the 22 common skin diseases analyzed.

### Development of online diagnosis app

**Fig. 4:**
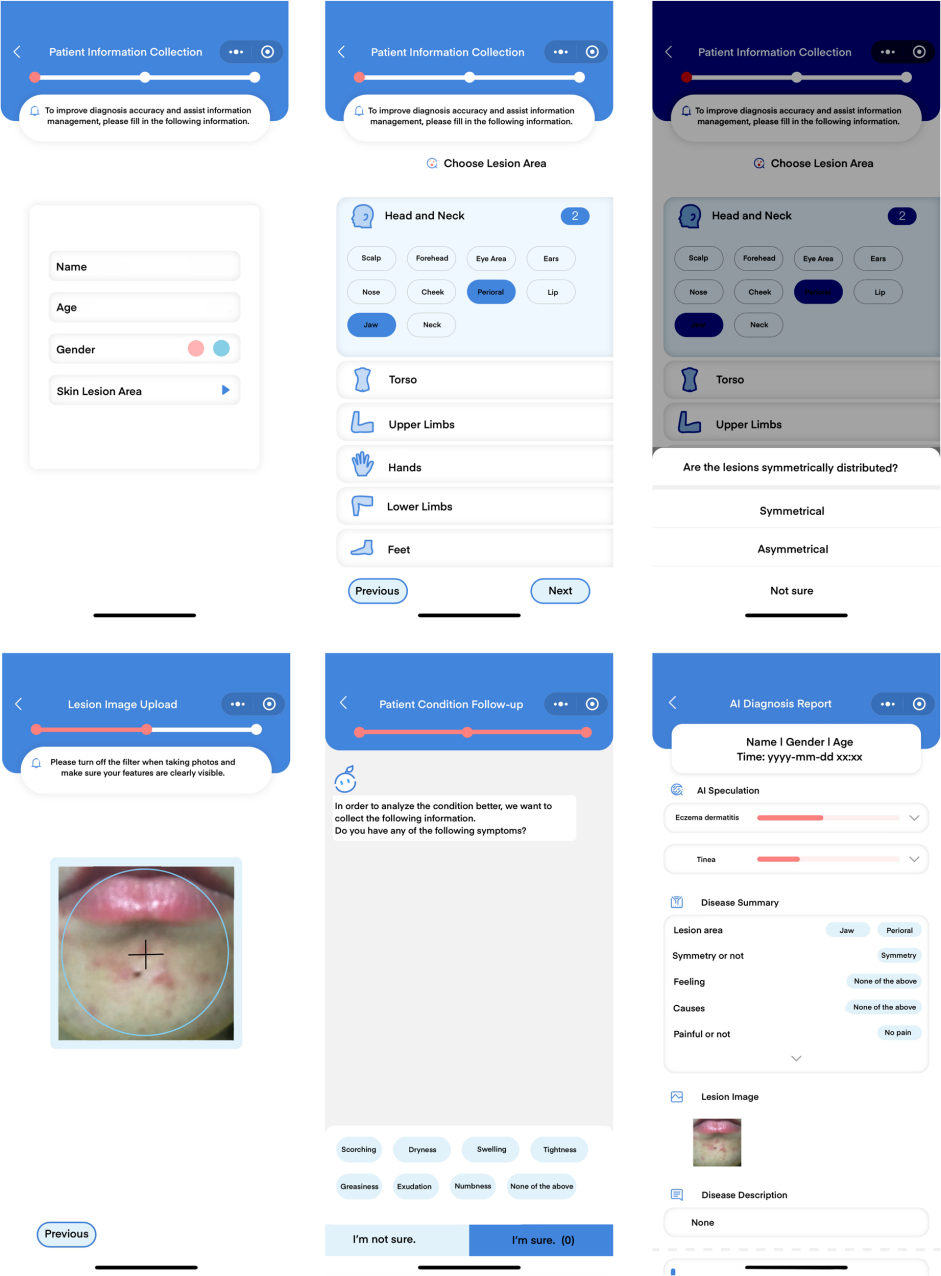
Screenshots of the app ‘Huifu’. Starting from the first line, patients information is collected to build a personal health record. Then, images of the lesion area are required to be uploaded. Our model is used to give a diagnosis based on the image uploaded. We use a follow-up system to further help improve diagnosis accuracy. Finally, the recommended diagnosis is presented.

We developed a prototype of an online dermatology diagnosis system implemented as a WeChat-based app named ‘Huifu’ following the JAMA CLEAR dermatology guidelines^25^, which could be used on smartphones, as shown in Fig. 4. We have included a completed CLEAR Derm checklist in Supplementary Table 1 to show our adherence to the guidelines. This app enables patients to receive diagnostic advice from our model by directly taking and uploading images from their smartphones. After collecting basic information from patients to build a health record, Huifu combined a survey and picture-taking of a skin lesion. The process begins by inquiring about the location of the lesions and their symmetry. Based on this information, the software guides the patient through different image collection protocols. We employ a pre-processing module to assess image clarity, lighting conditions, and camera distance to ensure that the images meet the collection standards. Subsequently, our model detects and segments the skin lesion. If monkeypox does not rank within the top 10 confidence levels, we proceed to request additional basic information related to the skin disease from the patient, such as whether it is accompanied by itching, pain, etc., to provide a more accurate diagnosis. However, if monkeypox does appear within the top 10 confidence levels, it will be considered a high-risk case. In such instances, we will pose specific questions, such as ‘Have you recently been in an area where monkeypox is prevalent?’ For cases with a high suspicion of monkeypox, a doctor will be assigned for a possible online consultation.

At current stage, our app has been served as a decision support system for clinicians to offer valuable suggestions and enhance the efficiency of doctors’ work. Since November 2021, we have been piloting the app with physicians in 18 tertiary hospitals, and it has assisted 186 doctors in making more informed diagnostic decisions. Lesion images are taken using smartphones, either by physicians or under their guidance, whereupon the images are uploaded for AI analysis. The final diagnostic judgment, however, rests with the physicians. We monitored backend usage data and requested feedback from doctors on the consistency of the model’s results with their own judgment. As shown in Supplementary Fig. 1, backend data was used to illustrate the consistency rate of top-2 diagnostic suggestions with physicians’ opinions, and the average using time during our test respectively. Based on our records up to November 2022, our app has been utilized in 26,676 patient encounters, with 21,288 completing the full diagnostic process. The average usage time per encounter stood at 107 s. Notably, the adoption rate of the app’s top-1 diagnosis by doctors is 63.04%. This is a significant metric for us, indicating that patients have achieved results at a faster pace than what would typically require more time from a doctor. It reflects the clinical utility and acceptance of our AI-driven diagnoses. In addition, the encouraging diagnostic performance, especially for emerging diseases such as monkeypox, has indicated the potential to collaborate with public health authorities to assist in internet-based screening efforts for such conditions.

### Performance on benchmark datasets

Similar to the case of monkeypox, our model holds promise for early malignancy detection, potentially leading to significant improvements in patient outcomes. However, in Chinese online forums there are few images related to malignancies (as discussed in Supplementary Information), potentially due to the relatively low incidence rate and limited awareness among Chinese Internet users^26^. Besides, it is also difficult to collect standard validation or test malignant images from our collaboration hospitals. Therefore, in this part we consider two benchmark datasets including related data: Fitzpatrick17k^27^ and diverse dermatology images (DDI)^28^ datasets. At the same time, we notice that our model was initially trained and tested on photos predominantly featuring East Asian individuals, potentially introducing some bias. These two benchmark datasets, gathered from countries beyond China, allow us to assess our model’s generalization across diverse races and skin tones.

We began by consolidating specific labels from benchmark datasets to align with our predefined labels. Given our primary focus on detecting malignant diseases, particularly melanoma as the most severe form of skin cancer, we isolated melanoma from the broader malignancy category, establishing two distinct classes: malignancies and melanoma. Consequently, we formulated a classifier encompassing 24 classes. Images that didn’t match these 24 labels were categorized as ‘others’. Supplementary Table 2 contains detailed information on the label merging rules and dataset sizes. Due to the inadequacy of the DDI dataset to form a new class for training, we opted to combine the two datasets together for this experiment. Classes with fewer than 10 images in each dataset are not considered, as such a small sample size can significantly skew the testing performance.

Firstly, we fine-tuned our model incorporating 50 images each from melanoma and malignancies into the filtered coarse labeled training set. Of these, 10 were sourced from DDI, and 40 from Fitzpatrick17k. Then we tested it on the remaining images, detailing the top-1 diagnosis accuracy for the classes in Table 2. Note that due to the dataset imbalance between the two benchmarks, our model demonstrated a tendency to perform better on Fitzpatrick17k. Although achieving a top-1 accuracy of 49.47% on our test set and showcasing a degree of general diagnostic capability on the benchmark dataset, the overall performance was underwhelming. The two newly introduced malignant classes exhibited relatively high diagnostic accuracy (12.50% and 16.79% on DDI, and 55.26% and 43.22% on Fitzpatrick17k). We attribute this disparity mainly to our model’s training on predominantly East Asian images, lacking exposure to diverse skin tones or races, while benchmark datasets are mainly non-East Asian images.

**Table 2:** Performance on the benchmark datasets.

	DDI		Fitzpatrick17k	
Top-1 Accuracy (%)	w/o 20 images	with 20 images	w/o 20 images	with 20 images
Acne			19.50	28.92
Actinic keratosis			28.21	45.27
Eczema dermatitis			7.56	31.15
Epidermal cyst	8.57	33.33		
Folliculitis			31.29	56.52
Lichen planus			21.38	30.57
Lupus erythematosus			5.01	40.08
Malignancies	16.79	40.15	43.22	48.86
Melanoma	12.50	27.27	55.26	59.96
Pigmented nevus	28.33	39.13	10.34	42.40
Psoriasis			17.56	45.92
Seborrheic dermatitis			34.36	47.83
Seborrheic keratosis	26.39	39.13	27.54	42.86
Urticaria			18.65	25.43
Viral warts	3.51	18.92		
Vitiligo			46.39	53.42
Others	20.54	24.86	24.85	30.95
Total except ‘others’	18.99	38.07	25.07	41.28
Total	19.45	33.69	24.95	35.57

We added 50 images from melanoma and malignancies into the filtered training set and created a classifier with 24 classes after label aligning. The fine-tuned models were tested on the remaining images of the benchmark datasets. Considering the racial difference between our training set and the benchmark dataset, the initial performance is not impressive. However, after we additionally extracted 20 images of each category from the benchmark datasets and added them into training, a great increase can be seen.

To address the knowledge gap within the model, we augmented the filtered training set with 20 images from each category, excluding malignancies and melanoma, sourced from the benchmark dataset. Due to data volume imbalances, we specifically added images from classes containing more than 50 images to ensure adequate numbers for testing. The diagnosis accuracy for the remaining benchmark images is detailed in Table 2. Remarkable enhancements were observed across nearly all classes. Overall performance on the DDI dataset increased from 19.45% to 33.69%, and on the Fitzpatrick dataset, it surged from 24.95% to 35.57%.

We attach the subgroup performance on both datasets in Supplementary Tables 3 and 4. On the Fitzpatrick17k dataset, we noted a gradual improvement in the model’s performance as skin tones deepened. Initially, the model achieved an overall performance of 33.13% for Fitzpatrick Scale I and 37.46% for Fitzpatrick Scale VI. We attributed this outcome to differences in the proportion and diagnostic accuracy of ‘others’ across varied skin tones. After excluding this class, the model’s overall performance elevated to 41.28%, demonstrating consistent performance across different skin tones (38.85%, 40.60%, 40.80%, 44.03%, 46.70% and 40.86% for Fitzpatrick scale I to VI). This improved performance across various skin tones suggests that our pre-trained model has captured key dermatological features, showcasing robust generalization ability. Additionally, its adaptability to malignant skin diseases underscores the potential for enhancing early intervention possibilities.

Specifically, the performance in identifying malignancies improved even without additional training data. On the Fitzpatrick dataset, the model’s accuracy in detecting melanoma reached 59.96% (sensitivity=0.59), matching other deep learning studies’ performance on the Fitzpatrick dataset^29^. The accuracy in identifying malignancies increased to 48.86%. This lower performance in malignancy identification might be because melanoma, as a specific disease, exhibits more distinct and concentrated features, while other malignancies present varied appearances. However, as malignancies is still quite a different class from the common diseases with our initial 22 classes, the performance is still outstanding considering the small amount of data needed, which indicates the potential in the early intervention of malignancies.

Additionally, we observed a significant decline in diagnosing acne compared to our test set. Upon image review, we believe this might be due to substantial morphological differences in acne across diverse skin tones and ethnicities, creating a gap that cannot be merely addressed by grouping it as the same ‘acne’ as present in our dataset. Similar to melanoma, if we extract 50 cases of acne from benchmark datasets as a new category, our model’s diagnostic accuracy on these acne images reaches 67.95%. Unfortunately, due to limitations in the quantity of benchmark datasets, it is challenging to further increase the number of images in the training set to explore the model’s diagnostic potential. However, our results emphasize that by considering the substantial disparities between the benchmark dataset and our training data, our model can be easily adapted to new downstream tasks with small number of training data.

### Bias discussion

Pre-training on images sourced from the Internet can carry and amplify social biases. In this section, we will provide an initial analysis of the biases present in our model. We address this issue from two perspectives: the data distribution in the training set and the performance across subgroups in the test set.

While naturally diverse, gathering metadata from the online images used to train the model is often challenging. To handle this, we conducted random sampling and manual labeling of the training set to showcase potential biases within our training dataset. We sampled 500 images from both the filtered and discarded coarse-labeled training sets, having trained dermatologists identify gender, age, and lesion area information in the corresponding images. Since labeling skin tones without professional training is difficult, we followed^27^ to compute the Individual Typology Angle (ITA) with YCbCr masks for skin tone estimation and derived Fitzpatrick scales. The unannotated data used for pre-training mirrors the unfiltered coarse-labeled dataset, a blend of these two subsets. The distribution of the sampled images is presented in Table 3. Remarkably, in the filtered image set, the percentage of ‘unknown’ for all subgroups, except for skin tone, was notably lower compared to the discarded image set. The proportion of unknown gender decreased from 47.80% to 29.34%, and unknown age reduced from 63.60% to 35.93%. In the filtered dataset, female account for the majority of images with explicit gender information, which may be because women are more active in seeking advice for skin diseases on online forums. The dataset predominantly concentrates on lighter skin tones, in line with the skin color distribution among East Asians. Additionally, compared to the discarded images, the proportions of the very light skin tone (I) and the dark skin tone (VI) decreased in the filtered images, indicating our filtering possibly eliminated images taken in extreme lighting conditions or through camera filters. The decrease in images with unknown information or under extreme lighting conditions partially indicates that our filtering approach retained images with more diagnostic information, potentially contributing to our model’s enhanced diagnostic performance.

**Table 3:** Meta Information distribution of the coarse-labeled training set.

		Images discarded	Images filtered
Gender	Female	25.80%	44.51%
	Male	26.40%	26.15%
	Unknown	47.80%	29.34%
Age	Infants	1.40%	1.20%
	Adult	29.20%	48.50%
	Prime	4.60%	12.18%
	The elder	1.20%	2.20%
	Unknown	63.60%	35.93%
Lesion area	Head/Neck	55.60%	62.08%
	Torso	10.20%	9.38%
	Upper extremity	5.00%	5.79%
	Hands	8.40%	5.19%
	Lower extremity	9.40%	5.59%
	Feet	5.20%	7.39%
	Unknown	6.20%	4.59%
Skin tone	I	71.00%	66.47%
	II	9.60%	12.97%
	III	9.20%	10.78%
	IV	3.60%	4.79%
	V	3.40%	3.59%
	VI	3.20%	1.40%

We sampled 500 images from both the filtered and discarded coarse-labeled training sets and annotated gender, age, and lesion area information. Skin tone was estimated by ITA scores. Other labels were obtained by human annotation. Apart from the skin tone, for all other subgroups, the percentage of ‘unknown’ in the filtered image set was significantly lower. The proportions of the very light skin tone (I) and the dark skin tone (VI) in the filtered images have decreased, which may imply a decrease in images taken under extreme lighting conditions. These results indicate the effectiveness of our filtering strategy.

As we collected patient metadata along with the validation and test set images from offline hospitals, we showcase subgroup performance on our test set in Supplementary Table 5. Specifically, we compared the performance of three models under distinct settings. Model A: pretrained on ImageNet dataset and finetuned with whole coarse-labeled set. Model B: pretrained on the dermatology dataset and finetuned with whole coarse-labeled set. Model C: pretrained on the dermatology dataset and finetuned with filtered coarse-labeled set with 50 validation images.

Notably, Model C outperforms both Model A and Model B. In subgroup analysis by lesion area, Model C significantly improved the diagnosis of conditions impacting the upper extremities, hands, lower extremities, and feet compared to Models A and B, while the improvement for head/neck and torso conditions was moderate. Across genders, all three models exhibited slightly better performance for females. The performance difference between males and females in the three models is 3.33%, 4.57%, and 3.72%, respectively. This indicates that Model C, without amplifying gender differences, exhibited an overall performance increase, emphasizing the filtering strategy’s capacity to generalize and provide balanced performance across genders.

Regarding skin tones, although a small amount of test images was categorized as Fitzpatrick scale V or VI based on their ITA scores, our test set comprises typical images of individuals from the Chinese population. Hence, rather than evaluating darker skin tone subgroup performance, we’re presenting the model’s performance facing East Asian skin that looks darker. We observed that Model C displayed relatively higher accuracy for skin types commonly found in East Asian populations, where the bias was most pronounced across all three models. Model C shows good consistency across Fitzpatrick scales II to V (52.58%, 49.40%, IV 57.98%, and V 51.98%, respectively). However, accuracy for the lightest (I) and darkest (VI) skin tones is comparatively lower, at 48.66% and 42.62%, respectively. Nonetheless, compared to Model A, the accuracy for skin classification in types I and VI is still higher by 6.87% and 4.05%, respectively. This might indicate that due to the uniqueness of the Chinese internet, the model’s diagnostic abilities for skin diseases among populations with the lightest and darkest skin tones are somewhat limited. It’s crucial to note that the difference in performance across various skin tone subgroups primarily stems from the inherent characteristics of the Chinese internet itself rather than from model training. Most internet users contributing to the dataset are East Asian residing with minimal immigration diversity in urban areas of China. While there are ethnic minorities in China with darker or lighter skin tones, these populations are primarily concentrated in border regions, resulting in lower representation within the dataset. Consequently, the dataset tends to represent individuals predominantly falling within Fitzpatrick skin types II to V, with significantly fewer representations of skin types I or VI. Our experiments on benchmark datasets further confirm this.

## Discussion

In conclusion, our study has revealed the value of unlabeled data on the internet for learning dermatological diagnoses using contrastive learning to pre-train a model. Although the improvement in diagnostic performance for 22 common skin diseases after fine-tuning was only 3% compared with the baseline model pre-trained on ImageNet, this should not be interpreted as a lack of value in the data from the Internet. The main reason for the limited improvement is that there are many labeling errors in the coarse annotations of data used during fine-tuning, which can greatly affect the model’s performance if not addressed properly. Therefore, we have established a training framework that uses the validation images as calibration to clean the coarse-labeled training set and maximize the value of the Internet data, resulting in a further significant improvement in the diagnostic performance of the model by a maximum of 4.6%, while reducing the computational costs required. It should be noticed that, although integrating ImageNet with unlabeled skin images offers a slight improvement in the pre-training stage, our filtering strategy can offer more performance gains, and emphasizes the necessity of a filtering approach when dealing with noisy datasets from the Internet. We demonstrate the ability of our model to transfer to downstream tasks with emerging diseases through the experiment of monkeypox. We also tested our pre-trained model on benchmark datasets to supplement missing out-of-distribution results in our test set and to verify our model’s generalization across malignant diseases and skin tones of different ethnicities. Our model can also distinguish malignancies with small amount of training data added. Due to utilizing data from the Chinese Internet, our model was trained predominantly on images of Asian individuals. While its initial performance on benchmark datasets primarily featuring other ethnicities may not be impressive, with the addition of a small number of images for guidance, it demonstrates excellent generalization on these new datasets as well. These results suggest that our framework has the potential for widespread use in the diagnosis of other skin diseases, particularly those for which labeled data is scarce.

Based on the prevalence of skin diseases and the pressure on medical resources, developing an effective AI-assisted diagnosis system for dermatological diseases can have significant value. Such a system can provide primary dermatologists and general practitioners with diagnostic expertise that is equivalent to that of top dermatologists, thereby bridging the gap between primary and advanced dermatology. A cross-sectional study^30^ has demonstrated that most dermatologists are willing to adopt AI tools to enhance time efficiency, diagnostic accuracy, and patient management. Additionally, for ordinary patients, online forums provide a platform to discuss their health concerns, including skin diseases. Using appropriate AI tools based on images allows patients to detect and treat potential diseases early. Timely diagnosis and treatment of rare and infectious skin diseases have significant clinical value, as they can encourage dermatologists to initiate appropriate treatment plans, improve patient experiences, reduce the risk of long-term sequelae, and reduce the incidence and mortality rates associated with severe skin adverse reactions or invasive skin cancer. At the macro level, it will contribute to the optimal utilization of medical resources, including targeted treatment and appropriate referrals to specialist physicians. This can alleviate the pressure on the healthcare system and minimize the waste of medical resources. Additionally, AI diagnostic information can be more directly and systematically integrated into other systems, providing information for public health interventions, policymaking, and resource allocation.

However, online skin disease diagnosis will face complex skin disease classification tasks with multiple disease subtypes and complex pathogenesis. Traditional supervised methods require a large amount of annotated data and may even involve human evaluation in the training process^31^, which is not feasible in the Internet context. Meanwhile, contrastive learning offers a potent tool for the automated diagnosis of skin diseases. Past uses of contrastive learning in the field of dermatology primarily involve structured and high-quality but limited-scale benchmark datasets. These datasets, geared for medical research, often concentrate on diseases with higher medical value and acquire images under stringent uniform capture requirements. Despite significant progress achieved by previous models in these specialized domains, the challenge arises for daily use to capture images that meet these stringent requirements and use these models^19–21^. For example, capturing images akin to the ISIC dataset using mobile devices can be quite challenging. Furthermore, these specialized datasets limit the types of diseases these models can cover, while skin diseases in daily life often exhibit a ‘long-tail’ distribution, demanding higher levels of coverage and generalization from models^32,33^.

Unlike the work in ref. ^34^ that improved the loss function to enhance the learning capability of contrastive learning for out-of-distribution (OOD) data representations, our work focuses on the effective utilization of web data right from the data source. These data sources often possess quality issues, making the research quite challenging. However, the inherent diversity of the internet data aligns better with regular people’s everyday scenarios, broadening the scope of our downstream tasks to support a more diverse set of common diseases than traditional contrastive learning. Through our filtering approach, our model achieved a significant improvement in diagnostic accuracy, demonstrating its effectiveness. Furthermore, experiments on benchmark datasets indicated the remarkable generalization ability of our model, swiftly adapting to and handling unknown data. In practical application, our research covers not only common skin diseases but also emerging ones, as showcased by our successful application of the model for early detection of monkeypox. Merely by incorporating an additional 50 images of monkeypox into the training process, our model attained an impressive 61.76% top-1 accuracy, showcasing its outstanding adaptability to emerging and evolving health challenges.

Considering that physicians have limited opportunities to diagnose based solely on image information, it would be unfair to directly compare the performance in this scenario. However, it is possible to assess the value of our work by comparing the results with similar studies. A previous study^3^ reported diagnostic accuracy rates of dermatologists, primary care physicians (PCPs), and nurse practitioners (NPs) as 63%, 44%, and 40%, respectively. The performance of our model is approximately comparable to that of PCPs. It is worth noting that the dermatology images sourced from the Internet encompass not only common skin diseases. While our target diseases share a substantial similarity with the aforementioned study, they also include less commonly seen diseases such as systemic lupus erythematosus, lichen planus, and blue nevus, with diagnosis accuracies below 40%. These diseases are more challenging to diagnose or resemble malignant skin diseases, often receiving more attention and discussion on the internet. If we solely consider skin diseases included in the aforementioned study, our average diagnosis accuracy reaches 52.57%. On one hand, the existence of images depicting rare or atypical skin diseases underscores the value of internet skin image data. On the other hand, it signifies the potential for monitoring rare or emerging epidemic skin diseases on the internet. Although the experiments indicate that due to data biases, our model exhibits higher performance among East Asian populations, given the severe shortage of dermatologists and PCPs in China, our ‘Huifu’ software holds substantial value. Our app aligns with the current shift towards patient-centered diagnostic approaches. Smartphone cameras evidently provide a convenient and swift means of dermatological consultation for a larger number of patients. This work showcases the potential of online health forums as valuable resources for medical research and the development of AI-driven diagnostic tools, paving the way for more inclusive and accurate healthcare solutions.

AI-assisted diagnosis of skin diseases has immense potential for further advancement, especially with recent breakthroughs in multimodal language models like ChatGPT^35^. These models possess the capability to process both image and text information, enabling quick access to accurate information about skin diseases for patients and healthcare providers, along with personalized responses to users’ inquiries and descriptions. Furthermore, the integration of text-based patient symptoms with image-based skin lesions, gathered from internet forums and other user data sources, can further enhance diagnostic accuracy. However, it is important to note that dermatological conditions often encompass numerous atypical cases. Relying solely on images for a preliminary diagnosis and then mechanically asking questions to differentiate them from common diagnoses would be time-consuming and may not yield accurate results. Building upon our work, it may be possible to improve the efficiency of consultations by employing a common language model that can better target differential diagnoses sharing typical lesion features. This approach could optimize the efficiency of consultations. For skin lesions located far away from clusters of other diseases, the reliability of the diagnosis would increase, requiring only a few follow-up questions, thereby further optimizing efficiency. With the advent of Internet hospitals, these models can even identify similarities between patients with similar symptoms or conditions. Thus, incorporating internet data can lead to more effective and efficient development of skin disease diagnostic models. As AI technologies continue to advance, we can expect even more exciting possibilities for AI-assisted diagnosis of skin diseases in the future.

## Methods

**Fig. 5:**
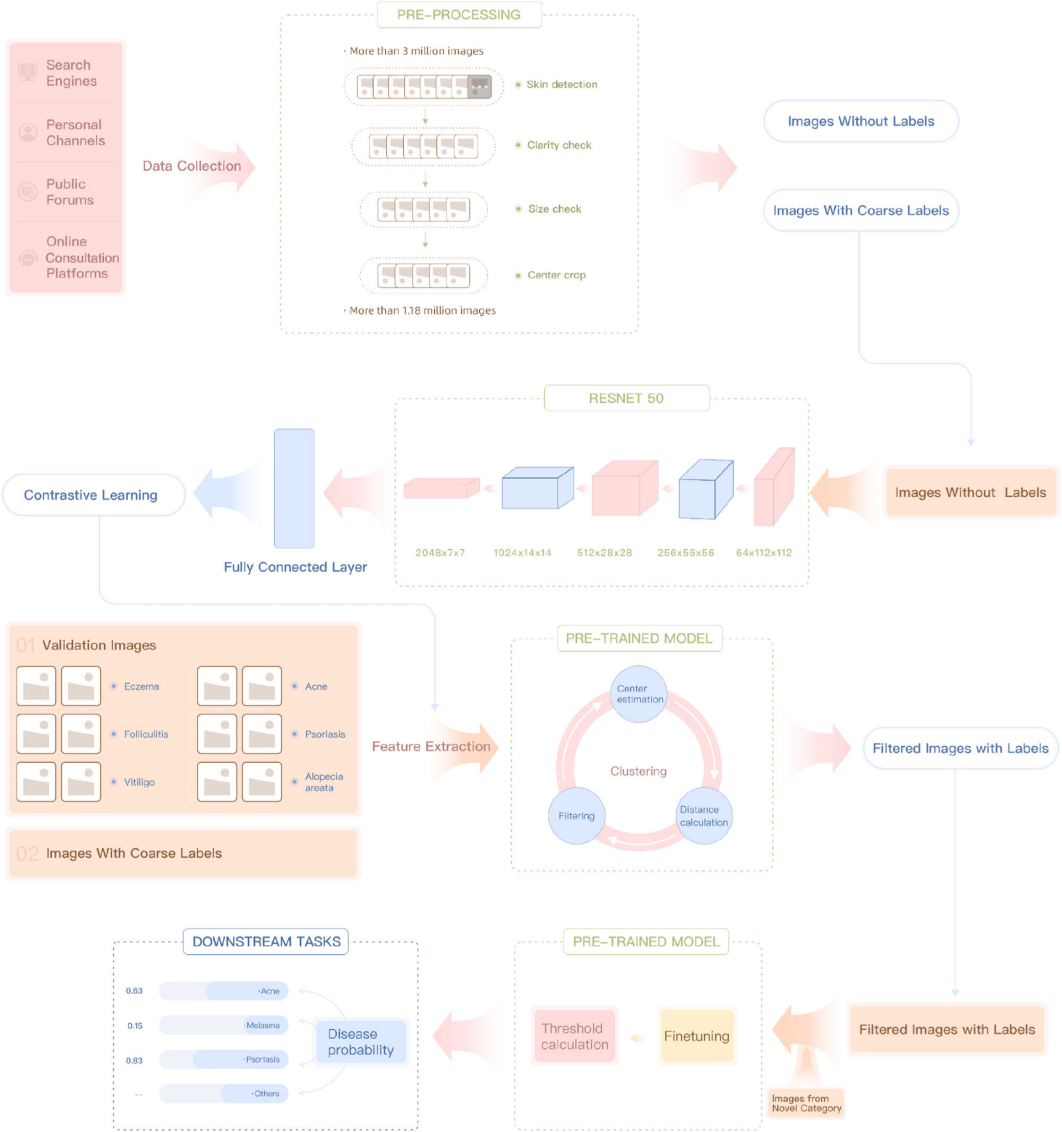
Overview of the proposed approach. Our approach is designed for online dermatological diagnosis scenarios and makes full use of the image data from the Internet. Starting from the top, firstly, images were collected from various Internet resources. Several screenings were implemented to pre-process the collected images. Besides 1.18 million images without annotations, 0.13 million images with coarse labels were obtained by matching keywords from topics and merged based on published standards. Secondly, a model was pre-trained on the unlabeled images using contrastive learning approaches, and it acted as a feature extractor later. Thirdly, features were extracted from both the coarse-labeled training set and the validation set. Clustering and filtering were performed to discard potential incorrect labels. Finally, the filtered training set was used to fine-tune the model, and a self-adaptive threshold was adopted to handle out-of-distribution images. The approach also allows a small number of images from new categories to be added for transfer to new downstream tasks, and it shows good generalization ability.

The overview of our framework is shown in Fig. 5.

### Data description

Significant progress has been made in the past few years based on large-scale annotated image datasets in object classification. The ImageNet Large Scale Visual Recognition Challenge (ILSVRC)^36^ has emerged as a central testbed for object classification research and has showcased landmark achievements in machine learning. For our experiment, we referred to the number of images used in the pre-training and fine-tuning of popular unsupervised learning methods trained on ImageNet and collected data from various sources on the Internet. The Medical Ethics Committee of the Third Affiliated Hospital of CQMU provided ethical review and approval for this study. Collaborating doctors obtained additional written informed consent using EC-approved forms from patients during the collection of validation and test images for the study. Our data collection process through the ‘Huifu’ app adhered to the user and content security solutions provided by the WeChat Mini Program, ensuring in-app informed consent was secured via a checkbox when gathering backend data.

The training images are collected from search engines, public forums, online doctor consultation platforms, dermatologists’ personal channels and other publicly available resources in China. We conducted searches based on specific keywords, allowing for a maximum of 3000 images up to date per search. This collection process was completed by the middle of 2021. Initially, we collected over 3 million images related to skin disease. To balance categories, we removed some samples from keyword subsets with sufficient image representation.

In the pre-process stage, to clean the data, we go through several screenings. We first apply a skin segmentation module using a U-Net^37^ to filter images without enough skin. We follow the experiment in ref. ^38^ and set the threshold as 0.75. We also drop images that are too small (less then 224 × 224), have extreme aspect ratio (over 3:1) or of low-quality as in ref. ^39^. At last, we use center-crop and resize all the images into 512 × 512 to create the training set of 1.18 million dermatosis-related skin images without annotations.

Note that annotation is unnecessary in the unsupervised pre-training phase of the model. However, labels are indispensable for fine-tuning it as a classifier. The skin disease images were annotated using two methods. Firstly, skin diseases or related terminologies were used as coarse labels for the images in self-organized communities and forums related to skin diseases in China. Secondly, skin disease images were directly searched on search engines, and databases with coarse labels created on the web were incorporated. To standardize and merge certain coarse labels, we relied on physician experience and dataset characteristics. We adopted industry-standard specifications such as CDISC^40^ and ICD10^41^ to transform large sections of unstructured text into standardized data through natural language processing techniques. For example, ‘whelks’, ‘pimples’, and ‘comedo’ were combined into the general category ‘acne’. We also merged labels that contained skin diseases that could not be reliably distinguished without consultation, such as ‘viral warts’ and those that contained only partial images that did not allow for identification of the lesion site, such as ‘tinea manuum’ and ‘tinea pedis’ into the broader category of ‘tinea’. Finally, we selected the 22 most representative skin diseases of the highest proportion, each with at least 800 images, for model construction, as listed in Table 4. The details of selecting these 22 diseases can be seen in Supplementary Information. In total, we collected 0.13 million dermatology-related skin images with coarse label information for fine-tuning.

**Table 4:** Data distribution for the coarse-labeled training set used in fine-tuning.

Disease Name	Number of Images	Disease Name	Number of Images
Acne	36942 (27.71%)	Lupus erythematosus	2515 (1.89%)
Actinic keratosis	822 (0.61%)	Melasma	5256 (3.94%)
Alopecia areata	1728 (1.30%)	Palmoplantar pustulosis	5970 (4.48%)
Androgenetic alopecia	1068 (0.80%)	Pigmented nevus	2149 (1.61%)
Blue nevus	1008 (0.75%)	Psoriasis	6483 (4.86%)
Cutaneous amyloidosis	6793 (5.10%)	Seborrheic dermatitis	16380 (12.19%)
Eczema dermatitis	11448 (8.59%)	Seborrheic keratosis	3437 (2.58%)
Epidermal cyst	4550 (3.41%)	Tinea	2116 (1.59%)
Folliculitis	900 (0.67%)	Urticaria	3868 (2.90%)
Herpes zoster	999 (0.75%)	Viral warts	4866 (3.65%)
Lichen planus	8672 (6.50%)	Vitiligo	5327 (4.00%)
Total		133297(100%)	

Despite 1.18 million dermatosis-related skin images without annotations used in the pre-training, we collected 0.13 million coarse-labeled images based on keywords and topics from the Internet. We relied on physician experience to standardize the labels referring to CDISC and ICD10.

To test the performance of our model, extra image dataset consisting of high-quality skin lesion images was obtained under standardized conditions and filtered to ensure quality to be used as validation and test set. Our validation and test set were gathered through collaboration with doctors in offline hospitals. Physicians physically examined the patients, assessed skin lesions, conducted interviews, and performed essential pathological tests to ascertain diagnostic results. After that, physicians captured the images using a smartphone in natural light or simulated natural light without any shadows and maintained a focal length of 1 while focusing on the center of the lesion. We disabled the camera’s beauty, whitening, smoothing, and filtering features during this process. For facial lesions, frontal and lateral images were captured at a 45° angle, while for symmetrical lesions on the extremities, two images of the affected area were combined. Asymmetrical lesions on the extremities required a complete image of the affected area. All images were required to ensure that the lesion covered 80% of the image area, and 20–30% of the surrounding skin was captured. In addition to this, physicians included essential meta-data (gender and lesion area) while photographing the skin lesions. The diagnosis for each case was determined by a dermatologist with at least 15 years of clinical experience in dermatology, and the dataset was reviewed by two dermatologists before inclusion. The dataset was created by 33 mid-to senior-level dermatologists. We prospectively collected 80 and 150 cases of each of the 22 most prevalent dermatological diseases respectively from 15 tertiary hospitals to serve as the validation and test set in the following experiments.

### Unsupervised encoder

We employed the formulation of swapping assignments between multiple Views (SwAV) presented in ref. ^15^ to learn the feature representations of skin diseases in an unsupervised manner using the online clustering method. SwAV encodes two different augmented views of the same image into features **z**_*t*_ and **z**_*s*_ respectively. Then a set of trainable code vectors ***q***_*t*_ and ***q***_*s*_ are computed by matching these features to a set of *K* prototypes $\left\{{c}_{1},\cdots ,{c}_{K}\right\}$. The similarity between these representations is formulated as a swapped prediction problem between positive pairs, whereby feature vectors from one view are forced to match the cluster’s code from the other view. The loss function is expressed with Eq. (1),

1 $$L\left({{\boldsymbol{z}}}_{t},{{\boldsymbol{z}}}_{s}\right)=l\left({{\boldsymbol{z}}}_{t},{{\boldsymbol{q}}}_{s}\right)+l\left({{\boldsymbol{z}}}_{s},{{\boldsymbol{q}}}_{t}\right)$$

where

2 $$l\left({{\boldsymbol{z}}}_{t},{{\boldsymbol{q}}}_{s}\right)=-\sum _{k}{{{\boldsymbol{q}}}_{s}}^{\left(k\right)}\log {{{\boldsymbol{p}}}_{t}}^{\left(k\right)},{{{\boldsymbol{p}}}_{t}}^{\left(k\right)}=\frac{\exp \left(\frac{1}{\tau }{{{\boldsymbol{z}}}_{t}}^{{\rm{T}}}{c}_{k}\right)}{\sum _{{k}^{{\prime} }}\exp \left(\frac{1}{\tau }{{{\boldsymbol{z}}}_{t}}^{{\rm{T}}}{c}_{{k}^{{\prime} }}\right)}$$

Notably, unlike other instance-based methods, SwAV does not employ negative pairs explicitly. Instead, the representation is prevented from collapsing through the batch-wise online code computations.

### Data cleansing based on distance

We train SwAV on images scraped from the Internet without annotation and use the model as a feature extractor for those images with coarse labels, which were subsequently used in the fine-tuning stage. The labels and the features of original training dataset are denoted as *Y*_train,i_ and ${Z}_{{\rm{train}},i}\in {{\mathbb{R}}}^{1\times d}\left(i=\mathrm{1,2},\cdots ,{N}_{{\rm{train}}}\right)$, with *d* dimensions for *N*_train_ samples. For the total *M* classes, we use a small subset of clean annotated data from doctor diagnosis as the validation set with *N*_val_ images for each class, the labels of which are denoted as ${Y}_{{\rm{val}},i}$ and use the pre-trained model to extract features ${Z}_{{\rm{val}},i}\in {{\mathbb{R}}}^{1\times d}\left(i=\mathrm{1,2},\cdots ,{N}_{{\rm{val}}}M\right)$ of them. These validation images have obvious inter-class distinctions can be served as a calibration for those images with label noise. Based on the assumption that each cluster for different diseases is a convex packet^42^, our strategy is to estimate the center of each cluster for every class based on the validation images.

3 $${C}_{{\rm{val}},i}=\frac{1}{{N}_{{\rm{val}}}}{\sum }_{j}^{{N}_{{\rm{val}}}}{Z}_{{\rm{val}},j}\;\text{for}\;{Y}_{{\rm{val}},j}=i,i=1,2,\cdots ,M$$

We calculate the Euclidean distance between the image in the training set and the center of the *M* clusters.

4 $${r}_{i,j}={{\rm{||}}{Z}_{{\rm{train}},i}-{C}_{{\rm{val}},j}{\rm{||}}}_{2},i=1,2,\cdots ,{N}_{{\rm{train}}},j=1,2,\cdots ,M$$

We discard the training images whose closest cluster does not match its coarse label. That is, we only retain images that meet Eq. (5).

5 $$\text{argmin}\left({r}_{i,\bullet}\right)={Y}_{{\rm{train}},\,i}i=1,2,\cdots ,{N}_{{\rm{train}}}$$

For dimension reduction, a principal component analysis is implemented. We project the feature representation of both the training set ***Z***_train_ and the validation set ***Z***_val_ to the vector space expanded by the first 100 principal components of the validation set.

### Transfer learning to downstream task with self-adaptive threshold

Collecting data and obtaining annotations from experienced physicians for rare diseases is a challenging task. In order to demonstrate the efficacy of our approach in handling rare diseases with small sample sizes, we gathered a dataset of 220 monkeypox images from various public websites on the internet. Out of these, 50 cases from literature case reports and the European CTC official website were selected as the training set, while the remaining 170 were used as the test set. To account for variations in ethnicity and skin color, we also collected 2146 skin images from English websites using relevant keywords such as Dermatosis, Skin diseases, Rash, Ringworm, Dermatitis, etc. These images were also included in the test set. Note that the recent monkeypox outbreak were first reported in 2022, which ensured these images will not be included in our pre-training dataset or coarse-labeled dataset. Also, manual screening ensured that the training and test sets of monkeypox images had no data overlap. The diverse skin disease images used were deliberately sourced from the Internet that dated after 2022. As a result, we expect no data leakage in this transfer learning experiment.

Since class categories or diagnoses that are not included in the algorithm’s training data are common in the online scenario, we needed to deal with images that belonged to out-of-distribution (OOD) classes. To address this, we employed a self-adaptive threshold to allocate images with low classification confidence to the ‘other’ class. After fine-tuning, we extracted the feature of the training set and calculated the distance among cluster centers of different classes.

6 $${R}_{i,j}={{\rm{||}}{C}_{{\rm{train}},i}-{C}_{{\rm{train}},j}{\rm{||}}}_{2},i=1,2,\cdots ,M,j=1,2,\cdots ,M$$

Taking the idea that the more distant the clusters, the easier it is to distinguish them, our approach considered the distance between the nearest and farthest clusters, with the threshold set as (7) to distinguish them effectively^43^.

7 $$T=\frac{\min \left({R}_{i,j}\right)}{\min \left({R}_{i,j}\right)+\max \left({R}_{i,j}\right)}$$

### Reporting summary

Further information on research design is available in the Nature Research Reporting Summary linked to this article.

### Supplementary information

Supplementary Information

Reporting Summary

## Data Availability

The entire dataset is not available due to privacy restrictions. However, part of the data that supports the findings could be available upon reasonable request from the corresponding author. The benchmark datasets used in this study could be found as follows: DDI (https://ddi-dataset.github.io), fitzpatrick17k (https://github.com/mattgroh/fitzpatrick17k).

## Code availability

Relevant code used in this study can be found on github https://github.com/shenyue-98/SwAVDerm.
