# Supplementary material for: Optimizing skin disease diagnosis: harnessing online community data with contrastive learning and clustering techniques
Source: NPJ Digit Med. 2024 Feb 8;7:28. doi: 10.1038/s41746-024-01014-x (PMC10853166; doi:10.1038/s41746-024-01014-x)
Supplement: Supplementary file 1 — Supplementary Information [file 41746_2024_1014_MOESM1_ESM.pdf]

Supplementary Information

Supplementary Figures

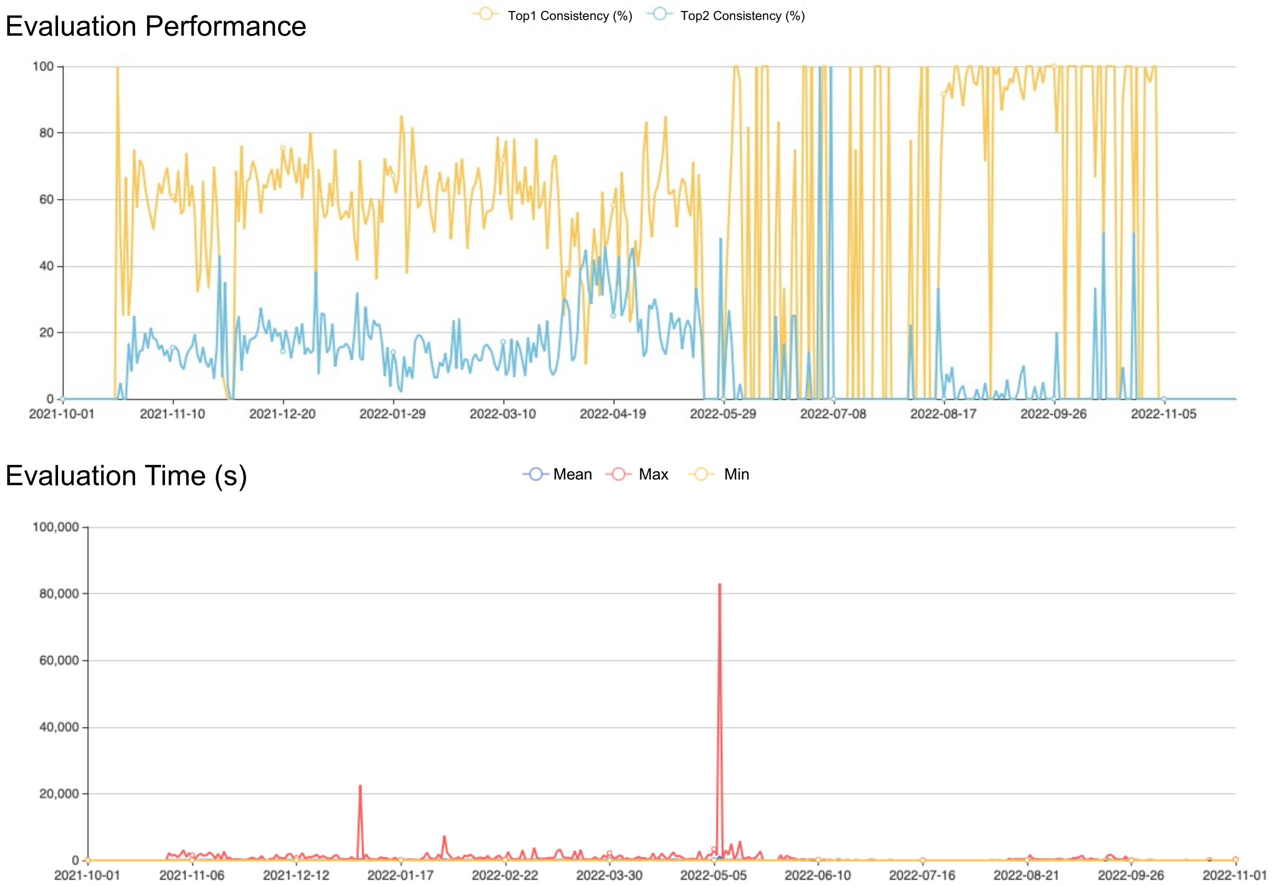

Supplementary Figure 1: Backend data of 'Huifu' app. Since November 2021, we have been piloting the app with physicians in 18 tertiary hospitals, and it has assisted 186 doctors in making more informed diagnostic decisions. Backend data was used to illustrate the consistency rate of top-2 diagnostic suggestions with physicians' opinions (upper) and the average using time (lower) during our test respectively. Based on our records up to November 1, 2022, our app has been utilized in 26,676 patient encounters, with 21,288 completing the full diagnostic process. The average usage time per encounter stood at 107 seconds. The adoption rate of the app's top-1 diagnosis by doctors is 63.04%.

Supplementary Tables

Supplementary Table 1: JAMA CLEAR-Derm checklists for our study

Checklist Item	Description	Compliance
**Data**		
Image types	Our model was trained using images sourced from online forums and initially tested on standard dermatology images collected from offline hospitals. In our 'Huifu' app, skin images are captured on smartphones.	Present

Image artifacts	The training images collected are assessed by a pre-processing module for clarity, lighting, and distance. Validation and test images are gathered under strict criteria.	Present
Technical acquisition details	When developing the model, images were sourced from the internet. In the 'Huifu' app, images are acquired through smartphone cameras. The app provides text-based reminders to users regarding appropriate lighting conditions and image clarity. It includes marker boxes to aid in lesion localization. Doctors assist patients in this process.	Present
Preprocessing procedures	The pre-processing module evaluates image quality before model training (see 'Data description' in Method Section). In our 'Huifu' app, if an uploaded photo doesn't meet the requirements, the app prompts patients to retake the photo.	Present
Synthetic images made public if used	Not used in our experiment.	Absent
Public images adequately referenced	Not used in our training process. We use benchmark datasets for testing our model's generalization ability to malignancies and out-of-distribution images (OOD).	Absent
Patient-level metadata	In the training set, metadata was hard to obtain since the data from online forums often lack such information. In our validation set, test set and 'Huifu' app, basic patient information (name, age, gender) and lesion details are collected to build a profile for each patient. But this information is not used for training our model.	Present
Skin tone information	Because the model was trained on images from Chinese online forums, and the app is tailored for Chinese users, our experimental scenarios are primarily focused on Asian skin tones. We used ITA scores to estimate skin tones for discussing bias.	Absent
Potential biases that may arise from use of patient information and metadata	We did not use patient information in our model.	Present
Dataset partitions	We created the unannotated pre-training dataset and coarse-labeled training set referring to the amount of ImageNet dataset. There is no leakage between train/test set.	Present
Sample sizes	The sample size employed in developing our model is detailed in 'Data description' in Method Section and Supplementary Methods. During our 'Huifu' app experiment, 26,676 individuals used the app, with 21,288 completing the testing process.	Present
External test set	See section 'Data description' in Method Section and Supplementary Methods for details of the validation and test set. No (OOD) images were gathered in these datasets. Nevertheless, we showcase the model's performance when dealing with OOD using the monkeypox experiment and benchmark datasets.	Present
Multivendor	Not used in our experiment.	Absent

images		
Class distribution and balance	See Table 4, Supplementary Methods and 'Bias discussion' in the Results Section for reference. We performed class balance before fine-tuning.	Present
Out of distribution images	The model will designate an image as 'others' if the classification confidence falls below the threshold. OOD images were not included in our test dataset. We assessed our model's performance in handling OOD scenarios through the monkeypox experiment and benchmark datasets. During our 'Huifu' app experiments, when the outcome is categorized as 'others,' doctors are expected to upload their opinions.	Present
**Technique**		
Labeling method	The labeling method employed during model development is detailed in 'Data description' in Method Section. In the 'Huifu' app, uploaded images are diagnosed by the model, and the results are subsequently reviewed by doctors.	Present
References to common/accepted diagnostic labels	See 'Data description' in Method Section.	Present
Histopathologic review	No relevant information was used in the training phase. Nonetheless, during the collection of the validation set, test set, and in testing on the 'Huifu' app, doctors confirm the diagnosis based on pathology and laboratory examinations.	Absent
Algorithm development description	The algorithm details can be seen in Method Section. The model generates results based on the uploaded image. Additionally, specific protocols are in place for high-risk cases, as observed in the instance of monkeypox, within the 'Huifu' app.	Present
**Technical Assessment**		
Public algorithm evaluation	We have presented the result of our model on DDI and Fitzpatrick17k dataset after selecting labels that match our scope, see 'Performance on benchmark datasets' in Results Section and Supplementary Table 3 and 4. Our 'Huifu' app can be accessed through WeChat for doctors to use.	Present
Performance measures	See Results Section for detailed analysis. We use our best model in our 'Huifu' app. Doctors will report whether the result from AI matches their diagnose.	Present
Benchmarking, technical comparison and novelty	See 'Evaluation of pre-trained models' and 'Performance on benchmark datasets' in Results Section and Discussion Section.	Present
Bias Assessment	See 'Bias discussion' in the Results Section.	Present
**Application**		
Use cases and target conditions	See 'Development of online diagnosis app' in the Results Section.	Present

Impact on healthcare team and patients	See Discussion Section.	Present

Supplementary Table 2: Label merging criteria of the two benchmark datasets and the number of images for each class.

DDI disease	Number	Fitzpatrick17k	Number	Ours
Acne cystic	1	Acne<br>Acne vulgaris	518	Acne
Actinic keratosis	4	Actinic keratosis<br>Porokeratosis actinic	358	Actinic keratosis
				Alopecia areata
				Androgenetic alopecia
Blue nevus	6			Blue nevus
				Cutaneous amyloidosis
Eczema spongiotic dermatitis	4	Allergic contact dermatitis<br>Eczema<br>Incontinentia pigmenti<br>Neurodermatitis	1362	Eczema dermatitis
Epidermal cyst	35			Epidermal cyst
Folliculitis	1	Folliculitis	342	Folliculitis
				Herpes zoster
		Lichen planus	491	Lichen planus
		Lupus erythematosus<br>Lupus subacute	519	Lupus erythematosus
Malignant diseases except melanoma	147	Malignant diseases except melanoma	1597	Malignancies
Melanoma<br>Melanoma acral lentiginous<br>Melanoma in situ	16	Malignant melanoma<br>Melanoma<br>Superficial spreading melanoma ssm	487	Melanoma
				Melasma
				Palmoplantar pustulosis
Epidermal nevus<br>Melanocytic nevi	120	Congenital nevus<br>Epidermal nevus<br>Naevus comedonicus	232	Pigmented nevus
		Psoriasis<br>Pustular psoriasis	706	Psoriasis

		Rosacea<br>Seborrheic dermatitis	227	Seborrheic dermatitis
Seborrheic keratosis irritated	72	Seborrheic keratosis	69	Seborrheic keratosis
Tinea pedis<br>Onychomycosis	3			Tinea
		Tick bite<br>Tungiasis<br>Urticaria<br>Urticaria pigmentosa	488	Urticaria
Condyloma acuminatum<br>Molluscum contagiosum<br>Verruca vulgaris<br>Wart	57			Viral warts
		Vitiligo	166	Vitiligo
Others	185	Others	8953	Others

Supplementary Table 3: Subgroup performance on the DDI dataset across skin tones.

DDI Top-1 Acc (%)	w/o 20 images				with 20 images			
Skin Tone	I/II	III/IV	V/VI	Total	I/II	III/IV	V/VI	Total
Epidermal cyst	12.50	0.00	7.14	8.57	20.00	25.00	50.00	33.33
Malignancies	26.32	15.87	8.33	16.79	32.50	42.64	44.44	40.15
Melanoma	16.67	0.00	20.00	12.50	66.67	25.00	0.00	27.27
Pigmented nevus	31.91	32.65	12.59	28.33	29.55	54.17	26.09	39.13
Seborrheic keratosis	13.33	26.32	43.48	26.39	30.43	50.00	63.16	39.13
Viral warts	0.00	0.00	9.52	3.51	22.22	0.00	18.75	18.92
Others	13.89	13.89	22.50	21.74	22.22	27.50	23.19	24.86
Total except ‘others’	19.28	18.32	21.59	18.99	30.08	45.59	38.46	38.07
Total	18.32	21.59	18.23	19.45	28.40	38.89	32.37	33.69

Supplementary Table 4: Subgroup performance on the Fitzpatrick17k dataset across skin tones.

Fitzpatrick Top-1 Acc (%)	w/o 20 images							
Skin Tone	I	II	III	IV	V	VI	-1	Total
Acne	8.94	19.14	27.71	36.07	15.15	0.00	0.00	19.50
Actinic keratosis	17.95	22.45	33.70	34.78	28.00	0.00	18.18	28.21
Eczema dermatitis	8.87	6.83	7.75	10.67	1.82	3.51	4.35	7.56
Folliculitis	30.00	39.18	31.31	25.49	25.81	55.56	12.00	31.29
Lichen planus	17.78	14.95	24.00	26.13	25.97	20.51	0.00	21.38
Lupus erythematosus	8.16	3.97	3.80	3.96	1.67	9.76	7.14	5.01
Malignancies	38.72	46.50	42.81	41.89	44.66	34.78	49.28	43.22
Melanoma	66.67	57.32	49.33	47.92	30.77	36.36	57.89	55.26
Pigmented nevus	12.50	15.00	7.69	5.56	9.09	0.00	0.00	10.34
Psoriasis	19.38	14.80	19.81	26.53	13.43	16.67	6.52	17.56

Seborrheic dermatitis	50.00	28.54	20.00	22.58	0.00	0.00	50.00	34.36
Seborrheic keratosis	26.67	22.58	37.50	40.00	40.00	25.00	0.00	27.54
Urticaria	24.21	18.62	18.37	20.00	10.53	6.25	8.33	18.65
Vitiligo	28.57	43.75	44.12	52.38	53.85	47.62	0.00	46.39
Others	23.42	23.19	23.02	24.81	30.12	32.94	30.52	24.85
Total except 'others'	24.71	25.04	26.26	26.79	23.24	19.43	23.08	25.07
Total	24.04	24.11	24.45	25.64	27.55	26.84	27.21	24.95

Fitzpatrick Top-1 Acc (%)	with 20 images							
Skin Tone	I	II	III	IV	V	VI	-1	Total
Acne	25.22	29.06	36.59	37.29	13.33	0.00	0.00	28.92
Actinic keratosis	39.47	32.98	52.33	54.65	47.83	0.00	40.00	45.27
Eczema dermatitis	31.25	30.33	31.10	39.33	20.37	24.56	26.09	31.15
Folliculitis	51.85	67.78	58.51	45.83	80.00	55.56	37.50	56.52
Lichen planus	30.57	15.91	14.85	26.32	44.86	42.47	43.59	8.33
Lupus erythematosus	30.53	41.03	33.33	35.35	57.89	57.50	46.15	40.08
Malignancies	43.77	50.00	46.71	46.85	57.28	45.65	65.22	48.86
Melanoma	62.73	10.13	52.78	54.00	64.00	90.00	63.64	59.96
Pigmented nevus	35.00	45.45	41.67	41.18	61.90	60.00	16.67	42.40
Psoriasis	49.61	46.91	45.54	52.17	33.85	37.50	37.50	45.92
Seborrheic dermatitis	53.19	55.06	35.14	33.33	33.33	50.00	50.00	47.83
Seborrheic keratosis	50.00	40.00	25.00	50.00	80.00	33.33	0.00	42.86
Urticaria	30.00	21.58	27.72	31.71	25.00	6.25	12.50	25.43
Vitiligo	28.57	46.67	55.17	56.76	66.67	50.00	0.00	53.42
Others	28.05	28.63	29.69	34.10	34.31	34.69	39.94	30.95
Total except 'others'	38.85	40.60	40.80	44.03	46.70	40.86	41.63	41.28
Total	33.13	34.50	34.52	38.19	38.75	37.46	40.69	35.57

Supplementary Table 5: Comparison among subgroup performances for the three models.

Subgroup diagnosis accuracy (%)		Model A	Model B	Model C
Gender	Male	40.48	42.90	47.90
	Female	43.81	47.47	51.62
Lesion area	Head/neck	43.21	47.50	48.75
	Torso	43.75	47.88	52.76
	Upper extremity	39.79	39.05	49.11
	Hands	36.77	38.17	45.67
	Lower extremity	39.59	37.20	48.81
	Feet	46.65	48.56	58.63
Skin tone	I	41.79	44.37	48.66
	II	42.27	46.02	52.58
	III	43.03	45.15	49.40
	IV	46.06	48.81	57.98
	V	40.43	49.54	51.98

	VI	38.57	43.10	42.62
Total		42.05	45.05	49.64

Supplementary Methods

Collection of coarse-labeled training, validation and test set

The design and collection of the 22 diseases in our test set were inspired by the initial distribution of our coarse-labeled training set. During our data collection process, we evaluated coarse labels against stringent diagnostic criteria for skin diseases. In the early stages of our experiments, we started with a training set consisting of 231,493 cases. The distribution of the initial coarse-labeled data set is shown in Supplementary Figure 2. Notice that the primary out-of-distribution label is ‘hemangioma’ and ‘skin tumor’ (the first two yellow bars). Most of the images under these labels come from pathological tissue slides in popular science articles, as well as consultations and discussions on these types of diseases. There are not many images that meet our pre-processing scanning criteria. We excluded instances such as CT scans (the first red bar) that did not correspond to primary skin disease diagnoses. This resulted in a refined dataset of 205,771 cases. Notably, we found that 172,168 cases fell under the 22 major diseases and their subtypes, which accounted for 83.67% of the dataset.

Then we excluded malignant diseases from this set, as collecting such images from Chinese online forums for training was very challenging for the reason discussed before. Similarly, we also omitted data related to scars and tattoos because these conditions are typically discussed in the realm of aesthetics rather than medical diagnosis. After excluding these images, our set of 22 diseases covers 91.12% of the images. Despite the coarse-label training set used for fine-tuning still needs pre-processing to discard low-quality images, our selection for validation and test set was driven by this distribution. The primary objective was to validate the knowledge obtained from online images against real-world hospital scenarios. Diagnostic performance on a well-defined in-distribution test set aids in identifying areas for model improvement. Our validation and test set were gathered through collaboration with offline hospitals. Physicians physically examined the patients, assessed skin lesions, conducted interviews, and performed essential pathological tests to ascertain diagnostic results. In addition to this, they followed our specific image-capturing criteria and included essential metadata (gender and lesion area) while photographing the skin lesions. Subsequently, two additional doctors reviewed both the images and metadata we collected. This stringent process was employed to maximize the accuracy and reliability of our test set.

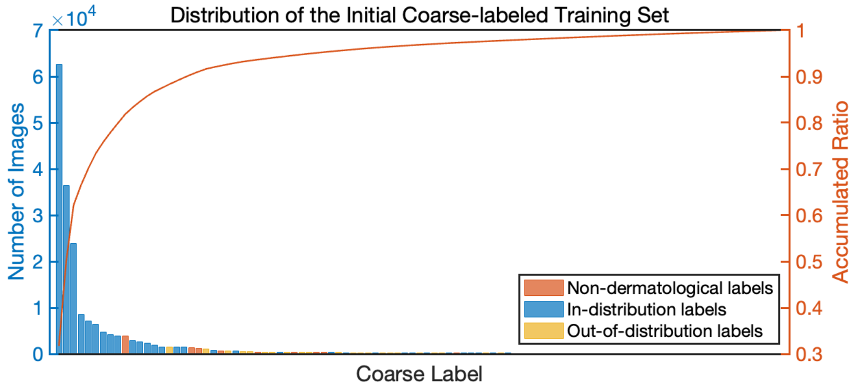

Supplementary Figure 2: Distribution of coarse-labeled data collected from the Internet. Initially we found that 172,168 cases fell under the 22 major diseases and their subtypes, which accounted for 83.67% of the dataset. Upon excluding non-dermatological labels and certain labels with insufficient images that met the

training criteria, our selected set of 22 diseases covered 91.12% of the images. This selection process also influenced the collection of the validation and test sets.
