# Supplementary material for: Optimizing skin disease diagnosis: harnessing online community data with contrastive learning and clustering techniques
Source: NPJ Digit Med. 2024 Feb 8;7:28. doi: 10.1038/s41746-024-01014-x (PMC10853166; doi:10.1038/s41746-024-01014-x)
Supplement: Supplementary file 2 — Reporting Summary [file 41746_2024_1014_MOESM2_ESM.pdf]

nature portfolio

Corresponding author(s): Junwei Lv, Zikun Wei

Last updated by author(s): Dec 28, 2023

Reporting Summary

Nature Portfolio wishes to improve the reproducibility of the work that we publish. This form provides structure for consistency and transparency in reporting. For further information on Nature Portfolio policies, see our Editorial Policies and the Editorial Policy Checklist.

Statistics

For all statistical analyses, confirm that the following items are present in the figure legend, table legend, main text, or Methods section.

n/a	Confirmed	
☐	☒	The exact sample size (*n*) for each experimental group/condition, given as a discrete number and unit of measurement
☐	☒	A statement on whether measurements were taken from distinct samples or whether the same sample was measured repeatedly
☐	☒	The statistical test(s) used AND whether they are one- or two-sided<br>*Only common tests should be described solely by name; describe more complex techniques in the Methods section.*
☐	☒	A description of all covariates tested
☐	☒	A description of any assumptions or corrections, such as tests of normality and adjustment for multiple comparisons
☐	☒	A full description of the statistical parameters including central tendency (e.g. means) or other basic estimates (e.g. regression coefficient) AND variation (e.g. standard deviation) or associated estimates of uncertainty (e.g. confidence intervals)
☐	☒	For null hypothesis testing, the test statistic (e.g. *F*, *t*, *r*) with confidence intervals, effect sizes, degrees of freedom and *P* value noted<br>*Give P values as exact values whenever suitable.*
☒	☐	For Bayesian analysis, information on the choice of priors and Markov chain Monte Carlo settings
☒	☐	For hierarchical and complex designs, identification of the appropriate level for tests and full reporting of outcomes
☒	☐	Estimates of effect sizes (e.g. Cohen's *d*, Pearson's *r*), indicating how they were calculated

Our web collection on statistics for biologists contains articles on many of the points above.

Software and code

Policy information about availability of computer code

Data collection	The training set was gathered from online sources using Scrapy. Both the validation and test sets were acquired from 15 tertiary hospitals, collected by a team of 33 mid-to senior level dermatologists. Benchmark datasets (Fitzpatrick 17k and DDI) are cited in the paper.
Data analysis	Python 3.9.7

For manuscripts utilizing custom algorithms or software that are central to the research but not yet described in published literature, software must be made available to editors and reviewers. We strongly encourage code deposition in a community repository (e.g. GitHub). See the Nature Portfolio guidelines for submitting code & software for further information.

Data

Policy information about availability of data

All manuscripts must include a data availability statement. This statement should provide the following information, where applicable:

- Accession codes, unique identifiers, or web links for publicly available datasets
- A description of any restrictions on data availability
- For clinical datasets or third party data, please ensure that the statement adheres to our policy

The codes and pre-trained model weights can be accessed at https://github.com/shenyue-98/SwAVDerm. Please note that the complete dataset is unavailable due to privacy restrictions. However, upon reasonable request, partial data supporting the findings may be made available by contacting the corresponding author. The Fitzpatrick17k and DDI datasets are publicly accessible.

Research involving human participants, their data, or biological material

Policy information about studies with human participants or human data. See also policy information about sex, gender (identity/presentation), and sexual orientation and race, ethnicity and racism.

Reporting on sex and gender	Sex and gender information were not utilized in the model's development. However, for bias analysis, a limited training set was manually annotated by human experts to assess the sex and gender distribution, as outlined in the paper. The sex and gender data for the validation and test sets were obtained from the collaborating hospitals.
Reporting on race, ethnicity, or other socially relevant groupings	The image datasets did not include race information. Nevertheless, considering that the training set was sourced from Chinese Internet forums and the validation/test sets were obtained from Chinese hospitals, it can be reasonably inferred that the majority of the images originate from the East Asian demographic.
Population characteristics	A subset of training images was annotated to analyze gender and age distributions, as detailed in the paper. Additionally, bias analysis was conducted to assess performance across various subgroups.
Recruitment	The validation and test set were gathered through collaboration with 15 tertiary hospitals involving 33 mid-to senior-level dermatologists. During the evaluation of our 'Huifu' app, we partnered with 186 doctors from 18 tertiary hospitals.
Ethics oversight	The Medical Ethics Committee of the Third Affiliated Hospital of CQMU provided ethical review and approval for this study.

Note that full information on the approval of the study protocol must also be provided in the manuscript.

Field-specific reporting

Please select the one below that is the best fit for your research. If you are not sure, read the appropriate sections before making your selection.

☒ Life sciences ☐ Behavioural & social sciences ☐ Ecological, evolutionary & environmental sciences

For a reference copy of the document with all sections, see nature.com/documents/nr-reporting-summary-flat.pdf

Life sciences study design

All studies must disclose on these points even when the disclosure is negative.

Sample size	For the unannotated training set, initially, we collected over 3 million images related to skin disease. After pre-processing, we created the training set of 1.18 million dermatosis-related skin images without annotations. We also collected 0.13 million dermatology-related skin images with coarse label information for fine-tuning. The validation set includes 80 images per class. The test set includes 150 images per class. Our test for the 'Huifu' app involved 26,676 patient encounters in total, with 21,288 completing the full diagnostic process.
Data exclusions	We discard images with low quality as described in the Method section. For the collection of validation and test set, all the images were strictly reviewed to meet our standards.
Replication	For the collection of validation and test set, all the images were strictly reviewed to avoid replication. We cannot avoid similar images in the training set since they were purely collected from the Internet, but this does not make any changes to our results.
Randomization	Participants were not allocated into experimental groups.
Blinding	Participants were not allocated into experimental groups. Group information was not needed for our model.

Reporting for specific materials, systems and methods

We require information from authors about some types of materials, experimental systems and methods used in many studies. Here, indicate whether each material, system or method listed is relevant to your study. If you are not sure if a list item applies to your research, read the appropriate section before selecting a response.

Materials & experimental systems

n/a	Involved in the study	
☒	☐	Antibodies
☒	☐	Eukaryotic cell lines
☒	☐	Palaeontology and archaeology
☒	☐	Animals and other organisms
☒	☐	Clinical data
☒	☐	Dual use research of concern
☒	☐	Plants

Methods

n/a	Involved in the study	
☒	☐	ChIP-seq
☒	☐	Flow cytometry
☒	☐	MRI-based neuroimaging

Plants

Seed stocks	Plants were not involved in this work.
Novel plant genotypes	Plants were not involved in this work.
Authentication	Plants were not involved in this work.
